# Comparison of oral conditions between the Macuxi and Yanomami indigenous ethnicities

**DOI:** 10.1590/1807-3107bor-2025.vol39.103

**Published:** 2025-10-10

**Authors:** Caio Vinicius Gonçalves ROMAN-TORRES, Richardson Mondego BOAVENTURA, Sergio Takashi KUSSABA, Doralice Severo da CRUZ, Wilson Roberto SENDYK, Debora PALLOS

**Affiliations:** (a) Universidade Santo Amaro – Unisa, Post Graduate Program in Dentistry. São Paulo, SP, Brazil.; (b) Faculdade Cathedral – Faces, Dental School, Boa Vista, RR, Brazil.; (c) Ministério da Saúde (BR), National Coordinator of Oral Health, Brasilia, DF, Brazil.

**Keywords:** Indigenous Peoples, Dental Caries, Periodontal Diseases

## Abstract

In the state of Roraima, Brazil, indigenous populations have distinct cultural habits and ethnic characteristics compared to those of the nonindigenous population. Changes in the oral health of indigenous people are critical areas of investigation for generating oral health indicators, which significantly influence the overall health of this population, given their fundamental role in the development of public health policies. Our objective was to compare the oral conditions of indigenous people of the Yanomamis (YANs) and Macuxis (MACs) ethnicities in the state of Roraima. A total of 148 indigenous people were evaluated: 83 (53.50%) YANs and 65 (46.50%) MACs. All indigenous participants were assessed at the Indigenous Health House (CASAI), in the municipality of Boa Vista/RR, and data regarding oral condition, DMFT index, periodontal indices, basic erosive wear examination (BEWE) index, and eating habits were obtained. Significant differences were found in the DMFT index, which was higher for MAC individuals than for YAN individuals (P<0.05). Periodontal evaluation revealed a significantly larger probing depth in YAN individuals aged over 35 years. Plaque index was higher for YAN individuals, with a significant difference noted in all compared groups. As for the BEWE index, no statistically significant difference was observed between YAN and MAC individuals. With respect to eating habits, YAN individuals consumed significantly more native foods, whereas MAC individuals consumed predominantly processed foods. Periodontal indices are affected by poor oral hygiene, and oral hygiene instruction and guidance are necessary and could help reduce the incidence of dental caries and periodontal disease.

## Introduction

National Oral Health Surveys (SB Brazil 2004 and 2010) demonstrated an improvement in the oral health status of the Brazilian population following the implementation of oral health policies. This improvement was due to the restructuring and reorganization of the guidelines of the National Oral Health Policy - Brazil Sorridente Program.^1^ Although ethnic differences were not addressed in the SB Brasil 2003 and 2010 surveys, the findings served as parameters for the reorganization of healthcare for indigenous peoples, resulting in enhanced service provision and considerable expansion of health teams in recent years.^2^ Nevertheless, contrary to expectations, a significant increase in the epidemiological indicators was observed among these populations. Despite the detection of worsening conditions and inequalities, preventive measures targeted at indigenous communities have been notably insufficient.^3,4^

One of the key factors contributing to the deterioration of oral health conditions in the indigenous population is the lack of training of most healthcare teams to overcome geographical, linguistic, and cultural challenges. Moreover, the fragmented structure of health service delivery is compounded by a lack of understanding of the different levels involved in the health/disease process.^5-9^

Studies conducted within various indigenous populations have shown comparable DMFT indices, even among different ethnicities, suggesting that regional and community-specific factors may substantially influence oral health indicators.^10-12^ The prevalence of untreated dental caries in indigenous adults is three times higher than that observed in nonindigenous adults, and the prevalence of periodontal disease is twice as high in indigenous people. The loss of all tooth elements is almost five times higher in indigenous populations, thus affecting their ability to eat, speak, smile, and socialize and having a negative impact on their quality of life.^4^

Variations in daily access to certain foods, such as sugar, can determine the oral characteristics of different ethnicities.^13^ Consumption of harder, unprocessed foods is associated with greater occlusal wear, whereas sugary foods can cause dental caries, indicating that dietary patterns can lead to oral health impairment.^3^ In some remote communities, the prevalence of dental caries is lower than that observed in many developed countries. In contrast, other communities with greater contact with nonindigenous groups, such as missionaries and miners, exhibit significantly higher DMFT indices than the national average.^14^

The limited number of publications employing robust methodologies on the oral health of indigenous communities, specifically those in the state of Roraima, demonstrates significant challenges to the strategic planning of oral health services. The few published studies further underscore the lack of comprehensive understanding of the oral health conditions among indigenous people.^15^

In a recent systematic review,^3^ the prevalence of dental caries ranged from 50% among indigenous people aged 18–36 months to 100% among those aged 65–74 years. The prevalence of periodontal disease ranged from 58% to 83%. The prevalence of malocclusion was 43%. Only two surveys with the Yanomami population were carried out in 1972 and 2008, and neither addressed the Macuxi ethnic group. Knowing the particularities of indigenous ethnicities is essential for developing effective care and prevention protocols. This study aimed to investigate, through clinical examination and statistical analysis, the differences between Macuxis and Yanomamis, both of which are predominant in the state of Roraima. DMFT indices, periodontal clinical parameters, and occlusal wear assessment were used to support the provision of primary healthcare services. Moreover, percentages of dental caries invasion provide epidemiological data for an operational plan in the region.

## Methods

The present study was approved by the president of the District Council of Indigenous Health (*Condisi*) and submitted to the National Research Ethics Council (*CONEP*) under process no. 1.998.028 and clinical trial registration no. NCT03660420. The study was carried out over a six-month period in two indigenous ethnic groups (Yanomami and Macuxi), which are predominant in Boa Vista, state of Roraima, by trained evaluators on scheduled days. All participants provided written informed consent in accordance with human ethics guidelines.

The indigenous people were evaluated at the Indigenous Health House (CASAI), located in the city of Boa Vista, during their medical appointments or while accompanying relatives who were being treated. Yanomamis were assessed at the Yanomami Health House and Macuxis were seen at the East Roraima Health House. Macuxis came from the municipality of Uiramutã, Maturuca base hub, currently consisting of 651 indigenous people. The Yanomanis came from the Awaris base hub with 1,529 individuals.

The participants were eligible for inclusion criteria if they were older than 18 years, lived near their community of origin, and identified as Yanomami or Macuxi. Individuals were excluded if they were edentulous, were older than 65 years, or refused to sign the informed consent form.

A total of 148 indigenous people aged 19 to 65 years were evaluated - 83 from the Yanomami ethnic group and 65 from the Macuxi ethnic group. They voluntarily agreed to participate in the evaluations, which were carried out between February 2016 and March 2017. Clinical examinations for decayed, missing, and filled teeth (DMFT index), as well as periodontal and occlusal wear analyses were performed on a convenience sample. Anamnesis was conducted with the help of an indigenous interpreter from both ethnic groups whenever necessary. The following data were obtained during the anamnesis: age, sex, previous oral hygiene instructions, frequency of oral hygiene, use of alcohol and tobacco, and consumption of native and processed foods. The questions were asked objectively. The frequency of oral hygiene was assessed by the number of times they brushed their teeth and used dental floss. Alcohol, whether processed or native, was regarded as frequent if consumed every day or more than three times a week. Smoking was considered frequent when used 10 or more times a day. Eating habits included the consumption of processed food — any items that were canned, bagged, or packaged industrially.

Data collection followed the methodology established by the SB Brazil 2010 survey (National Oral Health Survey)^1^ to ensure uniform interpretation of data and results across the various oral conditions assessed. The individuals were divided into the following age groups: Group 1: 19 to 34 years; Group 2: 35 to 44 years; and Group 3: 45 to 65 years. All teeth present were evaluated in the oral cavity, including third molars. The DMFT index was used for visual examination of dental caries on the occlusal, incisal, mesial, distal, buccal, lingual, and palatal surfaces of the teeth. The number of missing and restored teeth was also checked.

For periodontal and occlusal wear assessment, the individuals were divided into two age groups: those aged 35 years or less and those aged 35 years or more. The clinical periodontal parameters evaluated were probing depth (PD) and clinical attachment level (CAL) at six points per tooth. Plaque index (PI) and gingival index (GI) were evaluated at four points per tooth using a dichotomous scoring system.

The Basic erosive wear examination (BEWE) index was used to assess occlusal wear, recording the most severely affected surface of each sextant based on four risk levels, used for guiding clinical management. The four levels are: 0 = no surface wear; 1 = initial loss of enamel surface texture; 2 = hard tissue loss of less than 50% of the surface area; and 3 = loss of hard tissue greater than or equal to 50% of the surface area. All the teeth were evaluated, but only the highest index was considered; then, the highest indices of each sextant were added, and the result was obtained.^16^

The examiners were calibrated for periodontal clinical assessments, and both intraexaminer and interexaminer reliability was verified by calculating the standard measurement error and kappa coefficient (k>0.8). After clinical examination, participants were referred to the Cathedral College Dental Clinic for treatment of their oral conditions detected during data collection, according to each individual’s needs. Calibration for dental caries was performed in four stages, based on the methodology proposed by Peres et al.,^17^ yielding a kappa value of 0.86. For occlusal wear assessments, the evaluators were calibrated using images and clinical examination to verify reproducibility in the diagnosis of dental erosion. Calibration consisted of the examination of 10 individuals on two occasions, with a one-week interval between assessments. These individuals were not included in the main sample. Intra-examiner agreement yielded a kappa coefficient of 0.83.

The Mann-Whitney test was used to compare quantitative measurements between the two ethnic groups. Spearman’s correlation coefficient was also used to measure the correlation between ordinal and quantitative factors. The Mann-Whitney test was used to compare the means, and the Kolmogorov-Smirnov test (N≥30) was used to evaluate the normality of the data, and the assumption of normal distribution could not be confirmed. A significance level of 5% was adopted for all hypothesis tests. The SPSS V25 software was used for the statistical analyses.

## Results

A total of 148 male and female indigenous people aged 19 to 65 years were evaluated, among which 83 were Yanomamis and 65 were Macuxis (Table 1). According to the DMFT analysis (Table 2), statistically significant differences were observed between the ethnic groups as well as between men, particularly between older people (aged 36 to 65 years). In all these comparisons, Macuxis had significantly better outcomes than Yanomamis. Notably, among men aged 36 to 65 years, the mean DMFT index for Macuxis was 15.93 ± 1.42 compared to 8.53 ± 3.78 for Yanomamis (p value = 0.003).

**Table 1:** Distribution of ethnic groups, age group, and sex.

Variables	n	%	p-value
Ethnicity			
Macuxi	65	43.9	0.036
Yanomami	83	56.1	
Age group			
From 19 to 35 yrs	95	64.2	< 0.001
From 36 to 65 yrs	53	35.8	
Sex			
Female	77	52.0	0.485
Male	71	48.0	

**Table 2:** CPOD index for Macuxi and Yanomami, sex, and age.

Variables	Mean	Median	Standard deviation	Q1	Q3	N	CI	p-value
All								
MAC	12.08	12.0	5.82	8.0	16.0	65	1.41	< 0.001
YAN	8.34	7.0	7.46	2.0	14.0	83	1.61	
Female								
MAC	11.94	11.5	6.41	7.3	16.0	34	2.16	0.069
YAN	9.05	8.0	7.83	1.5	16.0	43	2.34	
Male								
MAC	12.23	13.0	5.18	9.5	16.5	31	1.82	0.001
YAN	7.58	7.0	7.07	2.0	11.0	40	2.19	
19–35 yrs								
MAC	8.59	9.0	4.52	5.0	12.0	37	1.46	0.094
YAN	7.45	6.0	6.93	2.0	11.0	58	1.78	
36–65 yrs								
MAC	16.68	17.0	3.79	14.0	18.0	28	1.40	0.004
YAN i	10.40	9.0	8.37	3.0	16.0	25	3.28	
Female								
19–35 yrs								
MAC	8.10	8.0	4.38	4.0	11.0	20	1.92	0.531
YAN	7.79	6.0	7.02	1.0	13.0	33	2.39	
36–65 yrs								
MAC	17.43	17.0	4.62	14.0	19.8	14	2.42	0.363
YAN	13.20	15.5	9.26	5.5	20.3	10	5.74	
Male								
19–35 yrs								
MAC	9.18	10.0	4.75	7.0	12.0	17	2.26	0.090
YAN	7.00	4.0	6.92	2.0	10.0	25	2.71	
36–65 yrs								
MAC	15.93	16.5	2.70	14.0	17.0	14	1.42	0.003
YAN	8.53	8.0	7.46	2.5	13.5	15	3.78	

MAC: Macuxi; YAN: Yanomami; DMFT: decayed, missing, and filled teeth; Q1: first quartile; Q3: third quartile; n: number of indigenous people; CI: confidence interval; yrs: years.

As for periodontal parameters (Table 3), a significant difference in PD was observed between Macuxi and Yanomami men, with mean values of 1.39 ± 0.13 mm and 1.64 ± 0.13 mm, respectively (p = 0.011). Among older men aged 36 to 65 years, the mean PD was also higher among Yanomamis (p = 0.024), but when older men were considered collectively, the mean PD was 1.31 mm for Macuxis and 1.87 mm for Yanomamis (p = 0.001). For CAL, a statistically significant difference between ethnic groups was observed only among individuals aged 36 to 65 years, with mean values of 2.25 mm for Macuxis and 2.93 mm for Yanomamis (p = 0.040). A difference was also found in the subgroup of men aged 36 to 65 years, with a mean age of 1.88 mm for Macuxis and 3.08 mm for Yanomamis (p = 0.023). In terms of gingival bleeding, a statistically significant difference was found between th e ethnic groups in all subgroups. Regarding plaque buildup, there was a difference between the ethnic groups in practically all subgroups, except for women aged 36 to 65 years (p = 0.266), for whom the difference between the ethnic groups was not significant. In other comparisons, the mean values were always higher for Yanomamis, i.e., 63.3 for Macuxis and 71.5 for Yanomamis (p < 0.001).

**Table 3:** Periodontal disease evaluated between both ethnic groups.

Variable	n	PD		CAL		GI		PI	
		Mean	p-value	Mean	p-value	Mean	p-value	Mean	p-value
Overall									
MAC	65	1.50 ± 0.38	0.515	1.94 ± 0.73	0.493	17.40 ± 8.3	0.361	63.30 ± 10.0	<0.001
YAN	83	1.56 ± 0.39		2.2 ± 1.05		21.80 ± 15.0		71.50 ± 10.2	
Female									
MAC	34	1.61 ± 0.35	0.104	2.11 ± 0.86	0.421	18.30 ± 10.5	0.674	63.60 ± 12.6	0.015
YAN	43	1.49 ± 0.36		2.06 ± 0.94		22.00 ± 16.7		70.40 ± 10.9	
Male									
MAC	31	1.39 ± 0.36	**0.011**	1.76 ± 0.51	0.061	16.50 ± 4.7	0.375	62.90 ± 6.2	<0.001
YAN	40	1.64 ± 0.42		2.35 ± 1.16		21.70 ± 13.2		72.70 ± 9.4	
19–35 years									
MAC	37	1.51 ± 0.37	0.68	1.71 ± 0.41	0.825	14.40 ± 4.5	0.495	61.10 ± 9.0	<0.001
YAN	58	1.47 ± 0.34		1.89 ± 0.83		19.80 ± 15.7		70.70 ± 10.8	
36–65 years									
MAC	28	1.5 ± 0.39	**0.024**	2.25 ± 0.93	**0.04**	21.40 ± 10.4	0.123	66.10 ± 10.7	0.005
YAN	25	1.78 ± 0.43		2.93 ± 1.17		26.60 ± 12.4		73.50 ± 8.5	
Female									
19–35 years									
MAC	20	1.56 ± 0.41	0.295	1.76 ± 0.45	0.633	14.50 ± 5.6	0.66	60.50 ± 11.5	0.011
YAN	33	1.44 ± 0.36		1.87 ± 0.89		20.80 ± 18.4		69.40 ± 11.1	
36–65 years									
MAC	14	1.68 ± 0.26	0.815	2.61 ± 1.06	0.77	23.70 ± 13.5	0.348	68.00 ± 13.2	0.266
YAN	10	1.64 ± 0.32		2.71 ± 0.82		25.80 ± 8.6		74.00 ± 9.7	
Male									
19–35 years									
MAC	17	1.45 ± 0.32	0.547	1.67 ± 0.37	0.336	14.30 ± 2.7	0.538	61.90 ± 5.0	0.001
YAN	25	1.51 ± 0.32		1.91 ± 0.75		18.40 ± 11.5		72.50 ± 10.3	
36–65 years									
MAC	14	1.31 ± 0.41	**0.001**	1.88 ± 0.64	**0.023**	19.00 ± 5.4	0.247	64.20 ± 7.5	0.003
YAN	15	1.87 ± 0.48		3.08 ± 1.36		27.10 ± 14.6		73.20 ± 7.9	

MAC: Macuxi; YAN: Yanomami; PD: probing depth; CAL: clinical attachment level; GI: gingival index; PI: plaque index.

Spearman’s correlation coefficient was used to measure the relationship between the daily frequency of oral hygiene and periodontal indices, including probing depth, clinical attachment level, gingival index, and plaque index (using the total sample). A significant correlation was obtained only between frequency of daily oral hygiene and plaque buildup, where r was - 0.447 (p < 0.001). This negative correlation showed that the higher the frequency of daily oral hygiene, the lower the plaque index, and vice versa. This correlation was classified as moderate (Table 4).

**Table 4:** Correlation between daily frequency of oral hygiene and periodontal indices.

Variable	Correlation (r)	p-value
PD	-0.068	0.414
CAL	-0.16	0.053
Gingival index	-0.136	0.099
Plaque index	-0.447	< 0.001

(r): Spearman’s correlation coefficient; PD: probing depth; CAL: clinical attachment level.

The BEWE index (Table 5) exhibited no statistically significant difference between the Macuxi and Yanomami populations in any of the subgroups. In terms of eating habits, differences were found in the type of food consumed by each ethnic group. As shown in Table 6, Yanomamis consume more native foods (67.9%) while the Macuxis have predominantly consume processed foods (84.8%).

**Table 5:** Comparison of ethnic groups in the different subgroups of the BEWE index.

Variable	Mean	Median	SD	Q1	Q3	n	CI	p-value
Overall								
MAC	8.82	8.0	4.10	6.0	12.0	65	1.00	0.480
YAN	9.54	9.0	4.17	6.0	12.0	83	0.90	
Female								
MAC	7.62	7.0	3.70	6.0	9.8	34	1.24	0.459
YAN	8.44	8.0	3.59	6.0	11.0	43	1.07	
Male								
MAC	10.13	12.0	4.16	6.5	13.0	31	1.46	0.779
YAN	10.73	11.5	4.45	7.0	13.0	40	1.38	
15–35 years								
MAC	6.73	6.0	3.57	5.0	8.0	37	1.15	0.052
YAN	8.02	7.0	3.38	6.0	10.8	58	0.7	
36–65 years								
MAC	11.57	12.0	2.99	9.0	13.0	28	1.11	0.231
YAN	13.08	12.0	3.67	11.0	16.0	25	1.44	
Female								
15–35 years								
MAC	6.10	6.0	3.49	3.8	6.3	20	1.53	0.121
YAN	7.39	6.0	3.04	6.0	10.0	33	1.04	
36–65 years								
MAC	9.79	9.0	2.89	7.0	12.0	14	1.51	0.111
YAN	11.90	11.5	3.14	9.5	12.8	10	1.95	
Male								
15–35 years								
MAC	7.47	7.0	3.62	5.0	9.0	17	1.72	0.184
YAN	8.84	8.0	3.68	6.0	12.0	25	1.44	
36–65 years								
MAC	13.36	13.0	1.82	12.0	14.5	14	0.96	0.688
YAN	13.87	13.0	3.89	12.0	17.0	15	1.97	

MAC: Macuxi; YAN: Yanomami; BEWE: Basic Erosive Wear Examination; sd: Standard deviation; Q1: first quartile; Q3: third quartile; n: number of indigenous people; CI: confidence interval; yrs: years.

**Table 6:** Eating habits of indigenous people evaluated.

Predominant diet	Yanomami		Macuxi		Total		p-value
	n	%	n	%	n	%	
Native	57	67.9*	10	15.2	67	44.59	0.027
Processed foods	26	32.14%	55	84.8*	83	55.41	0.008

n: number of indigenous people.

## Discussion

This study compared two important ethnic groups that differ in their relationship with the surrounding society: the Macuxis, who have a long-standing history of contact, and the Yanomamis, whose contact with society is much more recent. The extent to which this interferes with the indigenous way of life warrants investigation, mainly regarding the spread of diseases that are common among nonindigenous people and that did not traditionally affect more primitive and isolated groups way but are now spreading irreversibly. Therefore, it is essential to seek solutions to new and old problems caused by closer contact with nonindigenous populations. Interestingly, the Macuxi population lives in geographic areas with greater contact with nonindigenous people, which can influence the eating habits and oral hygiene of these indigenous people in the long term. According to our findings, the Macuxis invest in self-care practices related to toothbrushing, a behavior no longer observed among the Yanomamis, as they live in geographical areas farther away from the nonindigenous population.

Dental caries and periodontal diseases are the most prevalent oral conditions in nonindigenous populations and they are widely acknowledged as strong predictors of quality of life. In fact, several studies have demonstrated this correlation, but few studies have specifically addressed these conditions among indigenous populations, whose cultural practices and lifestyles are markedly distinct and diverse.^1,6,7^ Epidemiological studies using the DMFT index and periodontal indices based on nonindigenous populations are used to formulate public health policies aimed at indigenous populations. These policies, however, are inadequately tailored, causing many failures and waste of resources due to erroneous planning.^3,10,11,18^

Case studies^4,11^ involving the Xavante population in Brazil revealed a significant reduction in the incidence of dental caries in individuals aged 11 to 15 years, with the mean DMFT index decreasing from 4.95 in 2004 to 2.39 in 2007. This corroborates the findings of our study on the acceptance of traditional methods and preparation of the team to learn about and work with other cultures. A study with 288 individuals from four Xavante communities in Alto Xingu showed results similar to ours, despite examining slightly different but closely related age groups. The high mean DMFT index, especially in the group aged over 50 years, shows the neglect of health care for these populations.^19^

The indigenous Potiguara people in Paraíba,^20^ the Wai-Wai ethnic group in the Wakri community,^21^ and the Guarani indigenous people in Rio de Janeiro^22^ present dynamics of contact with the broader Brazilian society similar to that observed among the Macuxi. These studies revealed a low DMFT index for all age groups, with predominance of the decayed component, suggesting irregular and insufficient oral health care in these populations, with an emphasis on tooth extractions. The findings are very similar to those obtained for the Macuxi people, with a difference in the percentage of decayed teeth, perhaps due to the longer period of contact between the Macuxis and the surrounding nonindigenous society.

Yanomamis had a better DMFT index than Macuxis, which were significant in the 35-44- and 45-65-year age groups. This can be attributed to the proximity of the Macuxi population to nonindigenous people and to the isolation of the Yanomami population as they live in more remote communities. Moreover, the consumption of more processed foods by Macuxis and the consumption of more native foods by Yanomamis influence the DMFT index. In fact, the study by Freitas^18^ reported a very low DMFT index in the Yanomami population, such as 0.3 for individuals aged 19 years and 0.6 for those aged 35 to 44 years. Another study^11^ corroborating this finding in a Xavante population reported the deterioration of oral health due to proximity to the surrounding communities and the introduction of processed products, including a lack of preventive oral health programs.

Epidemiological studies of indigenous populations with poor oral hygiene^3,11,23^ showed an increase in gingivitis and periodontitis, which corroborates our study in which higher rates of PD, CAL, GI, and PI were observed in Yanomamis. In relation to Macuxis, there was inflammation but no development of periodontal pockets, which was observed in both ethnic groups. GI was higher among Yanomamis than among Macuxis, which is corroborated by other studies^1,18^ carried out with indigenous and nonindigenous populations. GI increased with age, , but there was no significant difference between Yanomamis and Macuxis. Studies in Brazil ^14,23-26^ and other countries ^27-30^ have shown that there is a need to improve the oral health status of the indigenous population, including those living in urbanized areas (i.e., close to nonindigenous people) and those living in remote areas.

When asked about self-care related to toothbrushing, 145 (92.36%) participants reported brushing their teeth daily - 72 (85.71%) in the Yanomami population and 73 (100%) in the Macuxis population. A change in this practice has been observed over the years, as evidenced by the study carried out by Pereira et al.,^23^ which demonstrated that Yanomamis did not have oral hygiene habits at that time. The increased exposure to hygiene practices among nonindigenous people resulted from their proximity to geographic areas with nonindigenous populations,^11^ mainly due to increased access to Indigenous Health House (CASAI) oral health teams in their communities. These collective actions can influence long-term changes in habits and customs regarding oral hygiene in Yanomami and Macuxis populations.

According to our findings, alcohol consumption was reported by 83 (52.87%) of those who were surveyed, 49 (58.33%) of whom were Yanomamis and 34 (46.58%) of whom were Macuxis. A few studies have addressed the relationship between alcohol dependence and periodontal diseases, which are generally associated with a synergistic effect with smoking.^31^ Alcohol-dependent individuals are more susceptible to periodontal problems, which was corroborated by our study, as Yanomamis had worse periodontal scores than Macuxis. Note that both Macuxis and Yanomamis, despite maintaining their cultural traditions in the manufacture of their typical alcoholic drinks, are already affected by alcoholism due to their proximity to the nonindigenous population, thus making the use of distilled alcoholic beverages a concern. Tobacco use was reported by 79 (50.32%) of those who were surveyed, 65 (77.38%) among the Yanomamis and 14 (19.18%) among the Macuxis. Tobacco use through mucosal absorption was observed solely in the Yanomami population, reported by 39 individuals. Smoking has been identified in several studies as a risk factor for periodontitis,^32^ with higher levels of alveolar bone loss, tooth mobility, probing depth, and tooth loss resulting in greater severity among indigenous people from urbanized populations.^6^

Occlusal wear also affects the quality of life of these populations, especially those who eat more native foods without industrial processing.^13^ Tissue loss can affect teeth, increasing their sensitivity and causing pulp necrosis, pain, and loss of vertical dimension, eventually affecting the aesthetics of these individuals.^3,16,18^

The occurrence and pattern of tooth wear are closely associated with educational, cultural, dietary, occupational, and geographic factors in the population.^24^ Occlusal wear is related to the influence of the surrounding community. Comparing previous studies between indigenous people and urban society, there is a greater pattern of wear in isolated populations and with less tooth loss due to native food consumption and the use of teeth as auxiliary tools in daily tasks.^25^ A study on Yanomamis showed a marked age-related increase in tooth wear due to the consumption of native foods, which are very hard and would be beneficial for reducing scars and fissures. This wear reduces the size of the dental crown, increasing the “moment of strength,” which enhances chewing efficiency and provides better self-cleaning and a massaging effect on the gums.^24^ This corroborates our findings, as the increase in tooth wear was greater in Yanomamis than in Macuxis. Additionally, the DMFT index for Yanomamis was also lower than that observed among Macuxis, which may be related to their more traditional diet.

Studies evaluating the oral characteristics related to the peculiar habits of each ethnic group are scarce in the literature. There are several factors limiting the elucidation of the individual characteristics of indigenous populations, such as distance from urban centers, language, authorizations for access to indigenous communities, and lack of equipment for clinical assessment. The individual characteristics of each ethnic group are very important and decisive in the study of the oral health conditions of indigenous populations, underscoring the importance of educational and informational campaigns.

## Conclusion

Compared with Macuxis, Yanomamis live in more isolated geographic areas and have different diets and habits. This fact probably influenced the lower PI among the Macuxis due to the oral hygiene instructions they received, including a higher DMFT index and reduced occlusal wear due to an industrialized and softened diet. Educational actions in oral health should be in line with the diversities of both indigenous ethnic groups, periodontal indices are affected by poor oral hygiene, and oral hygiene instruction and guidance are necessary and could help reduce the incidence of dental caries and periodontal disease.

## Data Availability

The content will be available when the article is published.
